# Ebola virus–like particles reprogram cellular metabolism

**DOI:** 10.1007/s00109-023-02309-4

**Published:** 2023-03-24

**Authors:** Huaqi Tang, Yasmine Abouleila, Anno Saris, Yoshihiro Shimizu, Tom H. M. Ottenhoff, Alireza Mashaghi

**Affiliations:** 1 Medical Systems Biophysics and Bioengineering, Leiden Academic Centre for Drug Research, Leiden University, Leiden, The Netherlands; 2 Department of Infectious Diseases, Leiden University Medical Center, Leiden, The Netherlands; 3 RIKEN Center for Biosystems Dynamics Research, Osaka, Japan

**Keywords:** Ebola, Cellular metabolism, Endothelial cells, Macrophage polarization

## Abstract

**Abstract:**

Ebola virus can trigger a release of pro-inflammatory cytokines with subsequent vascular leakage and impairment of clotting finally leading to multiorgan failure and shock after entering and infecting patients. Ebola virus is known to directly target endothelial cells and macrophages, even without infecting them, through direct interactions with viral proteins. These interactions affect cellular mechanics and immune processes, which are tightly linked to other key cellular functions such as metabolism. However, research regarding metabolic activity of these cells upon viral exposure remains limited, hampering our understanding of its pathophysiology and progression. Therefore, in the present study, an untargeted cellular metabolomic approach was performed to investigate the metabolic alterations of primary human endothelial cells and M1 and M2 macrophages upon exposure to Ebola virus–like particles (VLP). The results show that Ebola VLP led to metabolic changes among endothelial, M1, and M2 cells. Differential metabolite abundance and perturbed signaling pathway analysis further identified specific metabolic features, mainly in fatty acid-, steroid-, and amino acid–related metabolism pathways for all the three cell types, in a host cell specific manner. Taken together, this work characterized for the first time the metabolic alternations of endothelial cells and two primary human macrophage subtypes after Ebola VLP exposure, and identified the potential metabolites and pathways differentially affected, highlighting the important role of those host cells in disease development and progression.

**Key messages:**

• Ebola VLP can lead to metabolic alternations in endothelial cells and M1 and M2 macrophages.

• Differential abundance of metabolites, mainly including fatty acids and sterol lipids, was observed after Ebola VLP exposure.

• Multiple fatty acid-, steroid-, and amino acid–related metabolism pathways were observed perturbed.

**Supplementary Information:**

The online version contains supplementary material available at 10.1007/s00109-023-02309-4.

## Introduction

The outbreak of Ebola in West and Equatorial Africa has heightened worldwide concern. Ebola virus (EBOV) is a negative-sense, single-stranded, enveloped RNA virus that belongs to the *Filoviridae* family and etiological agent of a critical, often lethal disease known as Ebola hemorrhagic fever (HF) [1, 2]. The hemorrhagic disease caused by EBOV is characterized by initial fever and malaise followed by gastrointestinal symptoms, bleeding, shock, and multiorgan failure with case fatality rates ranging from 25 to 90% [1–3]. The recent Ebola outbreak (2018–2020) in the Democratic Republic of the Congo has claimed 2299 lives and the number of cases exceeded 3300, making it second only to the largest Ebola epidemic in West Africa of 2014–2016, highlighting the continued re-emergence of this pathogen [4]. Even though the first recorded outbreak was in 1976, much of the pathogenesis of EBOV remains unclear.

Macrophages are known as early targets and play an important role in EBOV pathogenesis [5, 6]. Infection of macrophages by EBOV stimulates the abnormal production of proinflammatory and immunomodulatory cytokines and chemokines (cytokine storm), disrupting vascular permeability, and recruitment of additional susceptible cells to the site of infection, which facilitates the systemic dissemination of the virus and leads to the development of viral hemorrhagic fever syndrome [5, 7, 8]. Moreover, secreted viral proteins do alter the balance between pro- and anti-inflammatory cytokines produced by non-infected macrophage cells upon exposure [7]. This effect on non-infected macrophages together with other immune cells can facilitate viral dissemination, establish systemic inflammation, and trigger an excessive cytokine storm that is detrimental for survival [6, 7]. Noninfectious virus-like particles (VLP) expressing EBOV glycoproteins (GPs) and matrix proteins were found to significantly regulate the gene expression profiles of primary human macrophages [9]. However, macrophages are phenotypically heterogenous depending upon the type of challenge encountered and accordingly perform disparate functions in the human body that may impact the outcome of the host immune responses. The cells show significant plasticity and metabolic reprogramming has been reported for macrophages in various pathological conditions. Macrophages are generally skewed towards two polarization subtypes, namely M1 or classically activated macrophages, which are elicited by exposure to proinflammatory signals, associate with production of proinflammatory cytokines (IL‐1β, IL‐6, IL‐12, IL‐23, TNF‐α, etc.), support Th1 cell immunity [10], and mediate clearance of pathogens [11]; and M2 or alternatively activated macrophages, which are anti‐inflammatory and immunoregulatory macrophages, secrete high amounts of anti-inflammatory cytokines (IL-4, IL‐10, TGF‐β, etc.) to promote resolution of inflammation, contribute to wound healing, support Treg activation [12], and retain homeostasis [13, 14]. Considering the importance of macrophages as major initial targets of EBOV, the different polarization status of macrophages might well influence EBOV pathogenesis.

Vascular instability and dysregulation are hallmarks of the pathogenesis in Ebola HF [15, 16]. It is thought that endothelial dysfunction can be caused either by proinflammatory cytokines (e.g., TNF-α) released from EBOV-infected monocytes/macrophages, or directly following EBOV infection of endothelial cells (ECs) [17]. Additionally, EBOV GPs are regarded as crucial determinants in the induction of cytotoxicity and injury in ECs, and consequently contribute to vascular dysregulation [6, 17–21]. Mechanical disruption of endothelial layers has been directly linked to the Rho/ROCK pathway, and can be reversed by Rho/ROCK inhibitors [18]. However, whether the pathology is limited to mechanics or also involves metabolic reprogramming is not known. The link between mechanics and metabolism has been widely recognized [22]. However, there is limited understanding of endothelial dysfunction that develops during EBOV pathogenesis, in particular, studies that characterize the virally induced metabolites changes in ECs are scarce.

Recent studies showcase that viral pathogenesis is significantly associated with hijacking host metabolic systems [23]. Thus, exploiting altered host metabolism and metabolic pathways represents a new research avenue for understanding the pathogenic mechanisms and will provide new insights on key checkpoints for biomarker discovery as well as novel drug targets for therapy. Metabolites represent the end products of biochemical pathways and therefore provide the most downstream biological/phenotypic knowledge. Recently, metabolomics proved expedient in aiding in the efforts to understand virus-host interactions as it is capable of providing a direct snapshot of the host metabolic profile in response to infection [24–27]. Particular cellular metabolic pathways such as carbohydrate, fatty acid, and nucleotide metabolism, have been reported to be altered as a result to infection by different viruses [28]. Most likely, each viral species triggers unique host metabolic alterations [24]. As such, various host metabolic reprogramming is associated with Influenza virus [29], Adenovirus [30], Dengue virus [31], Zika virus [32], Enterovirus [28], and SARS-CoV-2 [33]. Yet, very little is known about host metabolic reprogramming as a result to EBOV.

Investigations regarding the metabolic alterations to ECs and macrophages triggered by EBOV are limited. To address this critical gap in knowledge, we studied the impact of Ebola VLP on ECs and the different polarization status of macrophages by untargeted cellular metabolomics using direct infusion-mass spectrometry (DI-MS). Here, we dissect the intracellular metabolite alternations of ECs and M1/M2 macrophages treated by Ebola VLP and show that Ebola VLP can trigger changes mainly in fatty acid-, steroid-, and amino acid–related metabolism pathways for the three cell types, in a cell specific manner. This study demonstrates the strength of metabolomics for biomarker discovery and elucidates the effect of virus on host cells.

## Materials and methods

### Cell culture

Primary human umbilical vein endothelial cells (HUVECs) were provided by Leiden University Medical Center. HUVECs were cultured in Endothelial Cell Basal Medium 2 (C‐22211; PromoCell) supplemented with Endothelial Cell Growth Medium 2 SupplementMix (C‐39216; PromoCell) and 1% penicillin–streptomycin under 5% CO_2_, 37 °C incubator. HUVECs between passages 3 and 4 were used in all experiments.

Peripheral blood mononuclear cells (PBMCs) were isolated from buffy coats of healthy blood bank donors after informed consent was provided. This study was approved by Sanquin’ ethical advisory board and in accordance with the declaration of Helsinki and according to Dutch legislation. Monocytes were isolated through density gradient centrifugation over Ficoll-Paque followed by magnetic-activated cell sorting (MACS) using CD14 microbeads (130–097-052, Miltenyi Biotec) and differentiated into M1 or M2 macrophages with 5 ng/ml of granulocyte–macrophage colony-stimulating factor (GM-CSF; 130–093-864, Miltenyi Biotec) or 50 ng/ml macrophage colony-stimulating factor (M-CSF; 130–096-489, Miltenyi Biotec), respectively [10]. Cells were cultured at 37 °C/5% CO_2_ in RPMI 1640 medium (31,870,025, Gibco) supplemented with 10% FBS, 2 mM l-alanyl-l-glutamine (GlutaMAX; 35050038, Gibco), and penicillin–streptomycin (35050038, Gibco). As quality control, macrophages were stained for surface expression of CD14, CD163, and CD11b acquired on a flow cytometry (BD LSRFortessa, BD Biosciences). Macrophage differentiation and activation status was determined by quantifying IL-12 and IL-10 secretion by ELISA following stimulation of cells in the presence of 100 ng/ml lipopolysaccharide (LPS) for 24 h [14] (Fig. S1).

### Sample preparation for mass spectrometry

ECs and M1/M2 macrophages were seeded in 6-well plates at a density of 0.9 × 10^6^ cells/well and either treated with 1 μg/ml Ebola VLP (ZEBOVLP-100, The Native Antigen Company) or Ebola VLP buffer only as control (400 mM NaCl, 20 mM Tris–HCl pH8, 10 mM sodium citrate). At 2 h post-treatment, which was based on our previous study and other literature [9, 18], cells were washed with 3 × PBS then ice-cold 0.7% NaCl and subsequently lysed by ice-cold 80% methanol. Lysates mixture was sonicated for 10 min at room temperature before centrifugation at 16,000 rcf for 10 min at 4 °C, after which the supernatants were extracted with twice liquid–liquid extraction procedure using ice-cold water/methanol/chloroform (1/1/1, v/v/v). The methanol/water (aqueous) phase and chloroform (organic) phase were evaporated separately and dried materials were stored at − 80 °C for subsequent DI-MS analysis.

### Mass spectrometry

Prior to mass spectrometry (MS) measurements, dried extracts were reconstituted in 25 µL of 50% MeOH and 2 mM ammonium formate buffer for the aqueous phase, while for the organic phase, extracts were reconstituted in 80% acetonitrile and 2 mM ammonium formate buffer. From each sample condition, aliquot of 2 μL was added to a platinum-coated glass capillary with 2–3-μm bore size (CT-1, Humanix). All reagents used in the ionization solvents were of LC–MS grade and were obtained from Sigma-Aldrich. The capillary containing the aliquot was then attached to a nanoelectrospray adapter (nano-ESI) that is connected to the Q-Exactive mass spectrometer (Thermo Fisher Scientific) used in the analysis equipped with a nanospray source (Nanospray Flex; Thermo Fisher Scientific) for subsequent analysis. The mass spectrometer used was previously calibrated using Pierce LTQ Velos ESI calibration solution (Thermo Fisher Scientific). Measurements were performed in positive ion mode. The distance between the capillary and the inlet of the instrument was set to 2 mm and the inlet capillary temperature was set to 200 °C. The spray voltage was chosen to be 1.5 kV, maintaining a spray current between 100 and 150 nA. The resolution was set to 120,000 full width half-maximum. For untargeted analysis, selected ion monitoring (SIM) mode was chosen, scanning from a range of 100 to 1000 m/z with increments of 20 m/z. This method ensures a higher dynamic range in addition to a low signal to noise ratios without giving in on mass accuracy, thus enhancing the number of detected metabolites [34].

### Data preprocessing

Data generated from the mass spectrometry measurements was transformed from Thermo Fisher Scientific’s raw proprietary format to.txt files containing centroided m/z peaks using an in-house script. The converted data was then imported to R statistical software to assess the data and to perform quality control checks. Peak alignment was done using Sciex MarkerView software and the aligned data was further processed in R. A mass defect filter [35] was applied to remove salt clusters and artefacts from the dataset. Next, peaks with signal to noise ratio (S/N) < 3 were removed. Subsequently, a blank and medium subtraction filter was applied to remove background peaks. Finally, data was normalized using log2 transformation and scaled using pareto-scale. The dataset was then divided into three groups: ECs treated and untreated with Ebola VLP, M1 macrophages treated and untreated with Ebola VLP, and M2 macrophages treated and untreated with Ebola VLP.

### Statistical analysis

To demonstrate the metabolic profiles across the different samples in a reduced dimensional space, a principal component analysis (PCA) and a partial least squares discriminant analysis (PLS-DA) were carried out using the R statistical software. PCA aims to reduce dimensionality linearly and creates a new set of vectors that maximize the variance explained, while a PLS-DA tries to discriminate between groups by maximizing the covariance among a specific outcome and a matrix of metabolites [36, 37]. PCA was performed with the prcomp function from the basic stats package in R. PLS-DA was carried out with the mixOmics package in R. After normality check, data was split into parametric and non-parametric sub datasets. To discern the difference in the metabolic profile done due to Ebola VLP, univariate analysis, namely, Welch’s *t*-test (on the parametric portion of the data) and Wilcoxon rank-sum test (on the non-parametric portion of the data), were carried out and results were visualized using a volcano plot. Peaks with more than 1 log2 fold change or less than − 1 log2 fold change and with an FDR-corrected *p*-value < 0.1 were selected as significant and visualized with a heatmap. For peak identification, the significant peaks were run through an in-house script that matches the peaks against different metabolic databases, i.e., Kyoto Encyclopedia of Genes and Genomics (KEGG, https://www.genome.jp/kegg/), Human Metabolome Database (HMDB, https://hmdb.ca/), and Lipid maps structure database (LMSD, https://www.lipidmaps.org/). This tool uses pathway topological analysis accounting for the impact of individual detected metabolites within a pathway. The analysis depends on the pathway structure and the position of a metabolite within a pathway, meaning that central or nodal positions of metabolites in a pathway will have a greater impact than marginal or isolated positions.

## Results

### Global metabolic changes in ECs/M1/M2 following Ebola VLP treatment

**Fig. 1:**
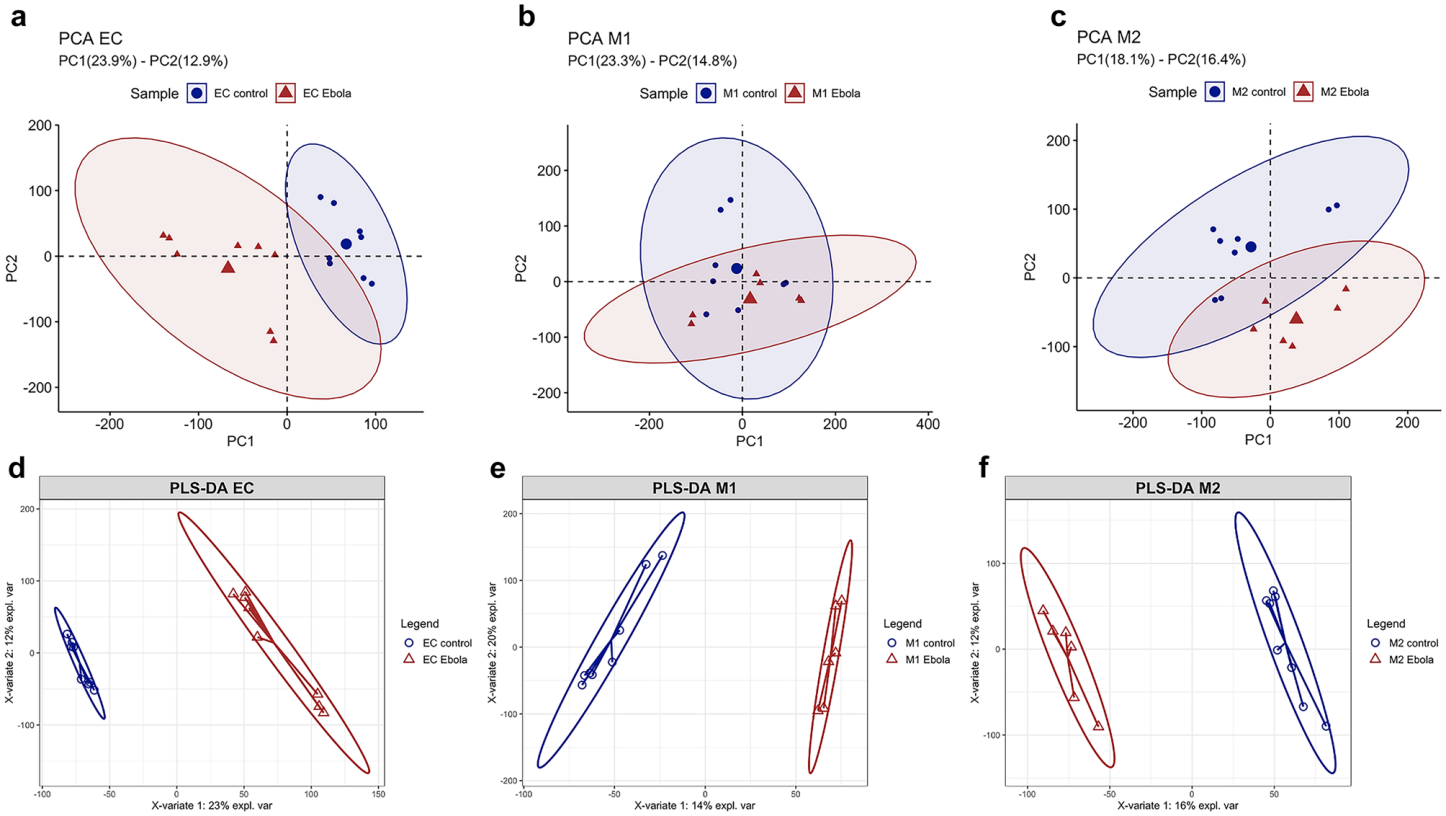
Principal component analysis and partial least squares discriminant analysis of ECs, M1, and M2 after treatment with/without Ebola VLP. The differences in the metabolic profiles between Ebola VLP treated vs. untreated ECs, M1, and M2 are shown in PCA (**a**–**c**) and PLS-DA (**d**–**f**), respectively. In both PCA and PLS-DA, red triangles represent cells cultured in the presence of Ebola VLP; blue circles are cells cultured without. Each triangle/circle corresponds to a sample

**Fig. 2:**
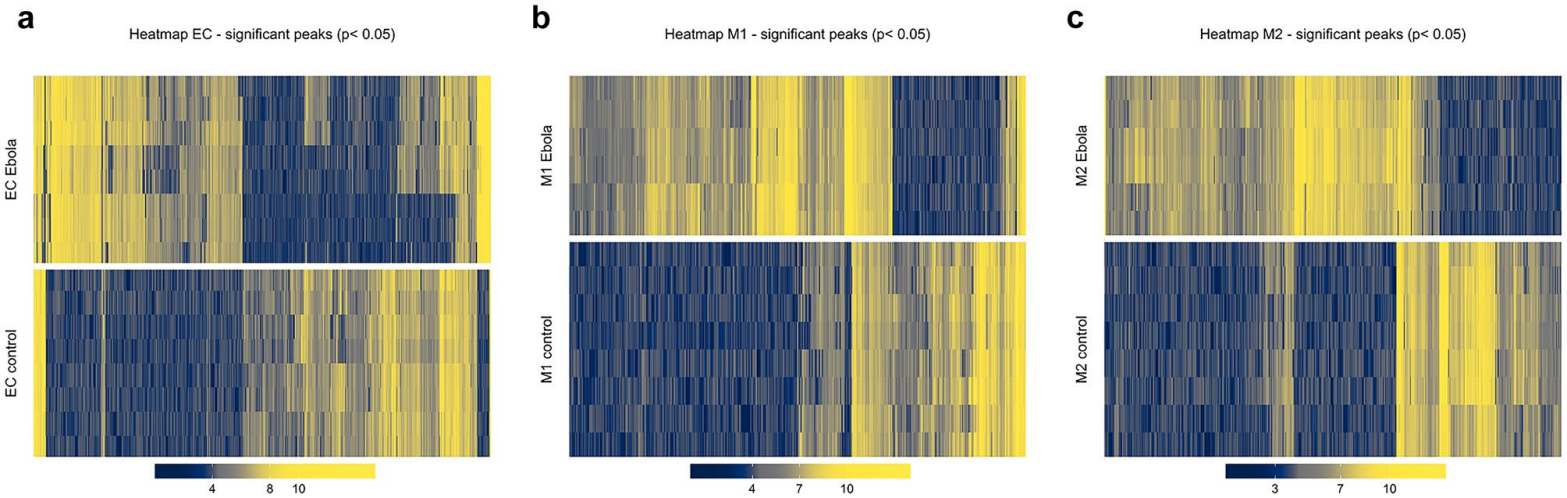
Heatmap of respective significant peaks found in ECs, M1, and M2 after treatment with Ebola VLP in comparison with untreated. Heatmap visualizing the intensity of each significant peak measured in ECs (**a**), M1 (**b**), and M2 (**c**). The color scales between dark blue and bright yellow represent lower intensity to higher intensity, respectively

To assess the possible metabolic alterations induced in ECs, M1, and M2 upon treatment with Ebola VLP, metabolomics data from samples were subjected to an unsupervised PCA followed by a supervised PLS-DA for visualization in a reduced dimensional space. The PCA plots show well-clustering behavior for both ECs and M2 groups (Figs. 1a, c and S2). However, M1 groups show a degree of overlapping as shown in Fig. 1b, which may indicate a similarity in the metabolic profile between the two conditions. Then, a supervised PLS-DA was performed and derived score plot revealed a clear clustering behavior (Fig. 1d–f), with specific metabolic profiles for each condition, in all the three groups. Next, Welch’s *t*-test and Wilcoxon rank-sum test were performed to narrow down peaks that are significantly different between the Ebola VLP treated and untreated conditions in each group. The distribution of significant and insignificant peaks can be visualized in the volcano plot (Fig. S3). Furthermore, a heatmap was created to visualize only the intensities of respective significant peaks with FDR-corrected *p*-value < 0.1 in every sample (Fig. 2). These visualized results provide an overview of the metabolic alternations in the three cell groups after Ebola VLP treatment; however, a more detailed analysis is necessary to better understand how Ebola affects cellular metabolism.

### Potentially altered metabolites and metabolic pathways in Ebola VLP–treated cells

**Fig. 3:**
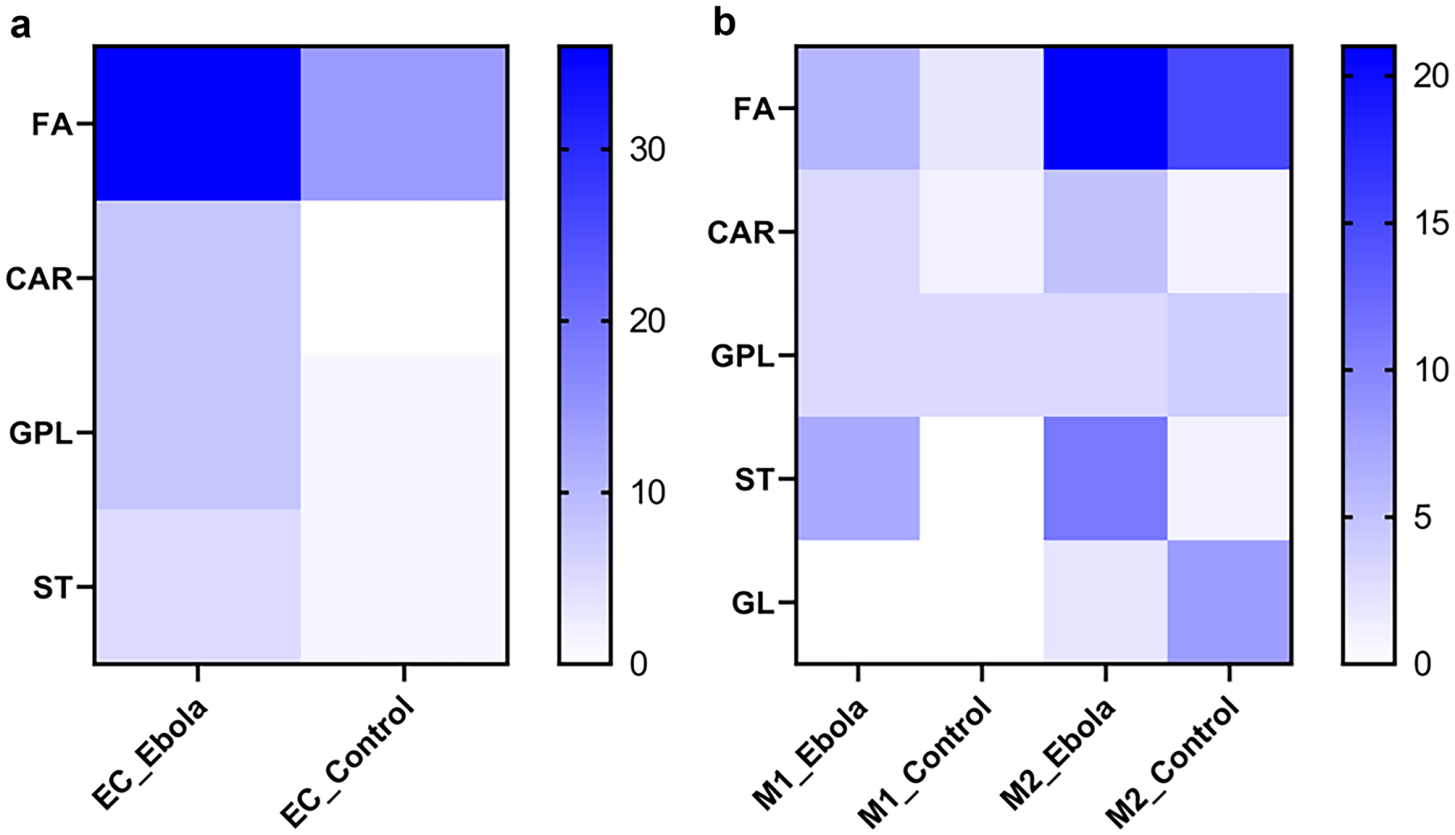
Distribution of lipid classes across ECs, M1, and M2 after treatment with Ebola VLP compared to control. The distributions of several lipid classes, namely, FA, CAR, GPL, ST, and GL for treated cells compared to their control, are shown in (**a**) and (**b**), respectively. GL was detectable in M2 only. The number of peaks matched in the database was counted; each hit corresponds to one peak encountered once. All peaks were filtered to abbreviation: CAR, acylcarnitine; FA, fatty acids; GL, glycerolipids; GPL, glycerophospholipids; ST, sterol lipids

To identify altered metabolites in response to Ebola VLP, the exact masses of the significant peaks resulted from the *t*-test were matched against databases, as mentioned in detail in the “Statistical analysis” section. A level 4 identification confidence could be reached to get unequivocal chemical formulas using the spectral information (e.g., adduct, isotope) [38–40]. Among the statistically significant peaks found, potential matches were identified. A summary of the putatively identified metabolites and lipids in ECs, M1, and M2 with *p*-value, adjusted *p*-value, chemical formula, class, and possible metabolic pathway for each compound is shown in Tables S1–S12, respectively. A color map for visualizing the distribution of lipid classes is shown in Fig. 3.

To gain further insight in the potential metabolic disruptions of Ebola VLP–treated cells, a pathway analysis was performed with respect to the significantly changed metabolites. A total of 7 pathways were observed to be potentially regulated in Ebola VLP–treated ECs, namely biosynthesis of unsaturated fatty acids, linoleic acid metabolism, arachidonic acid metabolism, glycerophospholipid metabolism, primary bile acid biosynthesis, glyoxylate and dicarboxylate metabolism, and valine, leucine, and isoleucine degradation. Meanwhile, alanine, aspartate and glutamate metabolism, arginine biosynthesis, histidine metabolism, glutamine and glutamate metabolism, glutathione metabolism, arginine and proline metabolism, purine metabolism, and pyrimidine metabolism pathways were observed in ECs without treatment (Table 1).

**Table 1:** Distinct metabolite signatures in ECs, M1, and M2 after Ebola VLP treatment

**Samples**	**Pathway**	**Hits**	**Metabolites**
**ECs_Ebola**	Biosynthesis of unsaturated fatty acids	2	FA 20:4 (arachidonic acid), FA 22:0 (docosanoic acid)
	Linoleic acid metabolism	5	FA 18:2 (linoleic acid), FA 20:4 (arachidonic acid), FA 18:2;O, FA 18:2;O3 (9,12,13,TriHODE), PC O-42:4 (lecithin)
	Arachidonic acid metabolism	3	FA 20:0;O (thromboxane), FA 20:4 (arachidonic acid), PC O-42:4 (lecithin)
	Glycerophospholipid metabolism	1	PC O-42:4 (lecithin)
	Bile acid biosynthesis	1	ST 27:1;O3
	Valine, leucine, and isoleucine degradation	1	FA 5:1;O
	Lysine degradation	1	FA 5:1;O
	alpha-Linolenic acid metabolism	3	FA 9:1;O, FA 18:3;O, PC O-42:4 (lecithin)
	Phospholipid metabolism	1	CDP-DG 25:0
	Valine, leucine, and isoleucine biosynthesis	1	FA 5:1;O
	Pantothenate and CoA biosynthesis	1	FA 5:1;O
	Fatty acid biosynthesis	5	FA 10:0, FA 10:1;O, FA 12:1, FA 12:1;O, FA 16:1;O
	Fatty acid degradation	1	FAL 16:0
	Steroid hormone biosynthesis	1	ST 21:3;O2 (progesterone)
**ECs_Control**	Alanine, aspartate, and glutamate metabolism	2	l-Aspartic acid, l-glutamic acid
	Arginine biosynthesis	2	l-Aspartic acid, l-glutamic acid
	Histidine metabolism	2	l-Aspartic acid, l-glutamic acid
	Glutamine and glutamate metabolism	1	l-Glutamic acid
	Glutathione metabolism	4	l-Glutamic acid, gamma-glutamylvaline, glutamyllysine, glutathione
	Arginine and proline metabolism	1	l-4-Hydroxyglutamate semialdehyde
	Purine metabolism	3	Deoxyadenosine monophosphate (DAMP), N-acetylneuraminic acid, N-acetyl-a-neuraminic acid, 2′-deoxyguanosine 5′-monophosphate, adenosine 2′-phosphate (AMP), 2-hydroxy-dAMP, adenosine 3′,5′-diphosphate, dGDP, ADP
	Pyrimidine metabolism	1	Dihydrothymine
**M1_Ebola**	Butanoate metabolism	1	FA 5:1;O2
	Pantothenate and CoA biosynthesis	1	FA 5:1;O2
	Ubiquinone and other terpenoid-quinone biosynthesis	2	2-Polyprenyl-6-methoxyphenol, ubiquinol
	Steroid hormone biosynthesis	2	ST 19:2;O2, ST 19:3;O2
	Steroid degradation	2	ST 19:2;O2, ST 19:3;O2
	Cysteine and methionine metabolism	1	3-Methyl-thiopropionic acid
	Glycerophospholipid metabolism	1	LPC 18:0
**M1_Control**	Citrate acid cycle	1	Oxoglutaric acid
	Fatty acid degradation	1	CAR 16:0 (l-palmitoylcarnitine)
**M2_Ebola**	Arginine and proline metabolism	1	l-Proline
	Pentose phosphate pathway	1	Deoxyribose
	Valine, leucine, and isoleucine biosynthesis	2	FA 5:0;O2, isopropylmaleic acid
	Pantothenate and CoA biosynthesis	1	FA 5:0;O2
	Arachidonic acid metabolism	5	Tetranor 12-HETE, FA 20:5;O, FA 20:4;O, FA 20:3;O (15(S)-hydroxyeicosatrienoic acid), FA 20:0;O (thromboxane)
	Steroid hormone biosynthesis	7	ST 19:2;O2, ST 19:1;O2, ST 19:0;O2, ST 19:3;O2 (androstenedione), ST 19:2;O3 (17beta,19-dihydroxyandrost-4-en-3-one), ST 19:4;O3, ST 18:3;O2(estradiol)
	Primary and secondary bile acid biosynthesis	1	ST 24:1;O4
	Pentose and glucuronate interconversions	1	Cholesterol glucuronide
	Linoleic acid metabolism	1	FA 18:2 (lineolic acid)
	Biosynthesis of unsaturated fatty acids	3	FA 18:2 (lineolic acid), FA 20:5 (EPA), FA 22:0 (behenic acid)
	Retinol metabolism	1	Retinoic acid
	Tryptophan metabolism	1	5-Hydroxyindoleacetylglycine
	Glycerophospholipid metabolism	1	LPC 20:4 (2-lysolecithin)
**M2_Control**	Steroid hormone biosynthesis	1	4-Methylpentanal
	Arginine and proline metabolism	1	1-Pyrroline-5-carboxylic acid, 1-pyrroline-2-carboxylic acid
	Alanine, aspartate, and glutamate metabolism	1	1-Pyrroline-5-carboxylic acid
	Valine, leucine, and isoleucine degradation	1	FA 5:1;O (3-methyl-2-oxo-butanoic acid)
	Isoprenoid/cholesterol biosynthetic pathway	1	Farnesol
	Steroid biosynthesis	1	ST 27:3;O
	Ether lipid metabolism	1	LPC O-18:0
	Glycerophospholipid metabolism	1	PC 35:4, PE 38:4
	Linoleic acid metabolism	1	PC 35:4
	alpha-Linolenic acid metabolism	1	PC 35:4
	Arachidonic acid metabolism	1	PC 35:4
	Sphingolipid metabolism	1	SM 40:1;O2

Similarly, the pathway analysis was also performed on M1 and M2 macrophages. The butanoate metabolism, pantothenate and CoA biosynthesis, ubiquinone and other terpenoid-quinone biosynthesis, steroid hormone biosynthesis, steroid degradation, and cysteine and methionine metabolism pathways were found modulated in M1 after Ebola VLP treatment, while the fatty acid degradation pathway was more evident in the M1 control group. Through the pathway analysis, we also confirmed a modulation of arginine and proline metabolism; pentose phosphate pathway; valine, leucine, and isoleucine biosynthesis; arachidonic acid metabolism; steroid hormone biosynthesis; primary and secondary bile acid biosynthesis; pentose and glucuronate interconversions; linoleic acid metabolism; biosynthesis of unsaturated fatty acids; retinol metabolism; tryptophan metabolism; and glycerophospholipid metabolism in the M2 Ebola VLP–treated group. Meanwhile, steroid hormone biosynthesis; arginine and proline metabolism; alanine, aspartate, and glutamate metabolism; valine, leucine, and isoleucine degradation; cholesterol biosynthetic pathway; steroid biosynthesis; ether lipid metabolism; glycerophospholipid metabolism; linoleic acid metabolism; alpha-linolenic acid metabolism; arachidonic acid metabolism; and sphingolipid metabolism were found more prominent in the M2 untreated group (for details, refer to the Tables S1–S12).

## Discussion

Experimental evidence for possible effects of EBOV on the metabolomes of affected cells has been scarce so far. With an untargeted metabolomics approach, in the current study, we show that exposure to Ebola VLP leads to metabolic changes in ECs and M1/M2 macrophages. Multiple pathways were also observed either upregulated or downregulated in each cell type.

Ebola VLP–treated ECs were mainly characterized by an alteration of lipids, fatty acids (FA), glycerophospholipids (GPL), sterol lipids (ST), and acylcarnitine (CAR) as can be seen in Fig. 3a. Previous analysis of plasma lipidome changes in Ebola-infected patients has revealed the essential role of lipid for the disease outcome and state, due to their function as membrane structural components, signaling molecules, and energy sources [41]. In agreement, we found an upregulation of FAs, especially involved in several FA metabolism pathways, namely FA 9:1;O, FA 10:0, FA 10:1;O, FA 12:1;O, FA 16:1;O, FA 18:2 (linoleic acid), FA 18:2;O, FA 18:2;O3, FA 18:3;O, FA 20:0;O (thromboxane), FA 20:4 (arachidonic acid), FA 22:0 (docosanoic acid), and FAL 16:0. There is substantial evidence that FAs have a critical role in mediating vascular ECs dysfunction via contributing to, for example, nitric oxide production, oxidative stress, inflammation, apoptosis, etc. [42, 43]. It has also been demonstrated that FA (e.g., linoleic, arachidonic, eicosapentaenoic, docosahexaenoic acids) can function as endogenous defense against a variety of invading microorganisms but at the same time can act as immunomodulating agents to cause inflammation [44, 45]. Among GPL, our results show that the expression of phosphatidic acids (PA; PA 40:4 and PA 44:4), phosphatidylcholines (PC; PC 39:0, PC 40:1, and PC O-42:4), phosphatidylethanolamines (PE; PE 29:1 and PE 42:0), and phosphatidylserines (PS; PS 37:2) is induced in Ebola VLP–treated ECs. PA is involved in the regulation of membrane dynamics (fusion and fission events) [46]; EBOV requires fusion of viral and cellular membranes mediated by Ebola GPs, which is solely responsible for virus-host membrane fusion machine required for virus entry into cells [47, 48]. In addition, PC is an important composite of cellular membranes [49]; a plasma lipidome study revealed PC level is significantly enhanced in Ebola infection survivors compared to fatalities [41]. Similarly, increase in PE plasma levels was reported as signature of EBOV infection [50]. In addition to FA and GPL, impaired steroid secretion is reported in EBOV infection [51]; however, the results here show an upregulation of ST in ECs after treatment. This may indicate the potential host defense function to counteract EBOV due to their ability of reducing inflammation and suppressing the increase in vascular permeability [52, 53]. Our data also shows that treated ECs have higher levels of CAR, which are essential intermediates for fatty acid β-oxidation and organic acid metabolism [54]. This tendency was also found in Dengue fever patients compared to healthy control in a previous serum metabolome study [55]. The elevated levels of CAR may indicate a disturbed energy metabolism in ECs upon Ebola VLP treatment.

A relative reduction of amino acids, purine nucleotides, and carbohydrate metabolites involved in multiple metabolic pathways was identified in Ebola VLP–treated ECs compared to non-treated. The amino acids found here have strong association with the metabolism of glucogenic amino acids (GAAs), including alanine, aspartate, and glutamate metabolism; arginine and proline metabolism; arginine biosynthesis; histidine metabolism; glutamine and glutamate metabolism; and glutathione metabolism. This may suggest that Ebola VLP–treated ECs display a drastic decrease in GAAs, which may reflect an upregulation of gluconeogenesis. Similarly, a decrease in the levels of most identified purine nucleotides and carbohydrates involved in purine metabolism, which are considered as a source of energy, was observed in treated ECs. The data may indicate a higher energy cost status in Ebola VLP–treated ECs, consistent with the results in a previous Ebola VLP–treated macrophages study [9].

Our results show that ST and FA are the two pre-eminent metabolite classes in both Ebola VLP–treated M1 and M2 groups. The change in ST metabolites is probably due to the involvement of steroid degradation and biosynthesis pathways in M1 and M2, respectively, according to our pathway analysis results. This is corroborated by a gene expression study that reported upregulation of the steroid hormone metabolism pathway in Ebola infected primary human macrophages [9]. Multiple studies have demonstrated that steroids have miscellaneous effects on the survival and phagocytic activity of macrophages, especially the polarization of pro-inflammatory M1 phenotype macrophages to anti-inflammatory M2 [56–59]. As for FA, they can be released and utilized for the formation of pro-inflammatory molecules that enhance the phagocytic action of M1 macrophages in the initial stages of microorganisms invading [44]. Most of the FA found in treated M2 are involved in arachidonic acid metabolism, which was also previously reported to be upregulated in the induction of M2 polarization [60]. Arachidonic acid is a key inflammatory intermediate and known to have a critical role in immune response; for example, it modulates the polarization and function of macrophages for anti-microbial subtype, inactivates enveloped viruses, inhibits fatty acid synthesis that is critical for microbes to survive, and aids in the resolution of inflammation [44, 61]. A recent clinical study also showed the elevated trend of arachidonic acid in the serum of primary Dengue-infected patients [55]. The upregulation of the arachidonic acid metabolism pathway detected in M2, may be responsible for elevating the inflammatory response, which is beneficial for host defense against EBOV. According to previous findings that M1 polarization of macrophages is able to inhibit EBOV infection while M2 polarization can enhance infection [5, 8], the ST and FA metabolites found here may contribute to retaining their polarization and function to counteract EBOV. This hypothesis is also supported by the evidence that these two classes of lipids were detected in lower levels in the M1 and M2 control group. Moreover, reduced levels of triacylglycerols (TG) were observed in treated M2 groups. TG is a class of lipid that plays an important antibacterial role and the inhibition of its synthesis negatively affects the production of proinflammatory cytokines and macrophage phagocytic capacity [62, 63]. This is consistent with the promoting feature of M2 for Ebola and the results found here. Additionally, dityrosine was detected in the treated M2 group, which is a biomarker for elevated levels of oxidative stress. In which, oxidative stress has an association with a decreased macrophage response capacity to pathogens [64, 65], this may further support the involvement of M2 in Ebola. While in Ebola VLP–treated M1 several lipids were detected, certain lipids, including LPC 18:0, 5,6-epoxy,18R-HEPE and N-arachidonoylglycine are reported as bioactive lipids. These bioactive lipids can be further metabolized to mediators that participate in the pro-inflammatory signaling and as a result contribute to a pro-inflammatory phenotype of circulating monocytes [66–69]. Such evidence may indicate that M1 macrophages tightly coordinate their metabolic programs to support immunological functions to counteract EBOV. In addition, platelet-activating factor (PAF) was observed in treated M1, which is a potent and versatile mediator of inflammation response to allergens and infectious processes, as well as the further recruitment of leukocytes and increase of vascular permeability [70]. This release of PAF from M1 was previously reported in macrophages obtained from Dengue-infected patients [71]. It was also proposed as a potential diagnostic and prognostic biomarker candidate for Lassa fever patients [72, 73].

It’s worth noting that this work serves as the initial step towards understanding the potential metabolic alterations done to host cells upon Ebola exposure; however, the study has certain limitations that will be addressed in the future. Ebola GP is a key mediator of Ebola pathogenesis and determinant of disease severity [74, 75]. It has been suggested to have a key role in activating endothelial cells directly and decreasing endothelial barrier function [76]. Studies also show that GP is critical for initiating a sufficient signal for the activation of macrophages [9, 77]. It is of interest to gain further insight how GP alone contributes to cellular metabolomic changes. The Ebola VLPs used in this work are composed of GPs, matrix proteins, and nucleoprotein, which are morphologically similar to the live virus and highly immunogenic, enabling us to have a comprehensive understanding of host cell response. Future complementary experiments (e.g., VLPs without GP, GP or matrix protein VP40 only) will contribute to better mechanistic understanding of the observed metabolic changes. While DI-MS has multiple advantages, mainly, high sample throughput capability, enhanced reproducibility, reduced time of analysis, and wide metabolic coverage making it best suited for untargeted metabolomics [78], its limitations also need to be taken into account. One limitation is the inability to distinguish between the structural isomers of certain metabolites. In addition, it is more susceptible to ion suppression due to the lack of a separation technique and it lacks sufficient evidence to confirm possible structural elucidation, especially in the case of lipids. Thus, further large-scale studies involving targeted and quantitative analytical platforms are required to confirm the metabolomic alterations as well as potential biomarkers detected using untargeted metabolomics studies. In addition, other omics approaches (i.e., transcriptomics and proteomics) can be applied to reach a more complete disclosure of the mechanisms by which EBOV can affect the host response that contributes to disease pathology. In addition, the Ebola VLP used here is composed of nucleoprotein, glycoprotein, and matrix proteins. Although it does not contain the viral genes necessary for replication, it is morphologically and antigenically similar to wild-type EBOV and has been proven to be useful tools to study early events of EBOV pathogenesis [77, 79]. It allows us to disentangle the contribution of viral proteins to host cells from the contributions of host immunity and the process of infection.

In summary, an untargeted metabolomics analysis was undertaken and found metabolite and metabolic pathway alterations that are involved in the Ebola VLP–induced cellular metabolic reprogramming to ECs, M1, and M2. These results may pave the way towards identifying potential biomarkers that can predict Ebola disease progression, severity, and outcome, which may be helpful for diagnostics efforts. In addition, upon further confirmation and more targeted analysis, the abovementioned metabolic pathways may represent potential candidate targets for future development of prophylactic or therapeutic countermeasures. Ultimately, these findings may expand our knowledge of EBOV-induced metabolic alterations at the cellular level and aid in further investigation on systemic alterations.

## Supplementary Information

Below is the link to the electronic supplementary material.

Supplementary file1 (DOCX 2321 KB)

Supplementary file2 (XLSX 11 KB)

Supplementary file3 (XLSX 12 KB)

Supplementary file4 (XLSX 10 KB)

Supplementary file5 (XLSX 14 KB)

Supplementary file6 (XLSX 10 KB)

Supplementary file7 (XLSX 9 KB)

Supplementary file8 (XLSX 10 KB)

Supplementary file9 (XLSX 12 KB)

Supplementary file10 (XLSX 11 KB)

Supplementary file11 (XLSX 12 KB)

Supplementary file12 (XLSX 11 KB)

Supplementary file13 (XLSX 14 KB)

## Data Availability

Important data generated or analyzed during this study is included in this published article. Further information is available from the corresponding author on reasonable request.
