# Supplementary material for: Ebola virus–like particles reprogram cellular metabolism
Source: J Mol Med (Berl). 2023 Mar 24;101(5):557–68. doi: 10.1007/s00109-023-02309-4 (PMC10036248; doi:10.1007/s00109-023-02309-4)
Supplement: Supplementary file 1 — Supplementary file1 (DOCX 2321 KB) [file 109_2023_2309_MOESM1_ESM.docx]

**Ebola virus-like particles reprogram cellular metabolism**

Huaqi Tang^1¶^, Yasmine Abouleila^1¶^, Anno Saris^2^, Yoshihiro Shimizu^3^, Tom H.M. Ottenhoff^2^, Alireza Mashaghi^1^*

^1^Medical Systems Biophysics and Bioengineering, Leiden Academic Centre for Drug Research, Leiden University, Leiden, The Netherlands

^2^Department of Infectious Diseases, Leiden University Medical Center, Leiden, The Netherlands

^3^RIKEN Center for Biosystems Dynamics research, Osaka, Japan

*Corresponding author

E-mail: [a.mashaghi.tabari@lacdr.leidenuniv.nl](mailto:a.mashaghi.tabari@lacdr.leidenuniv.nl)

^¶^These authors contributed equally to this work.

Journal name: Journal of Molecular Medicine


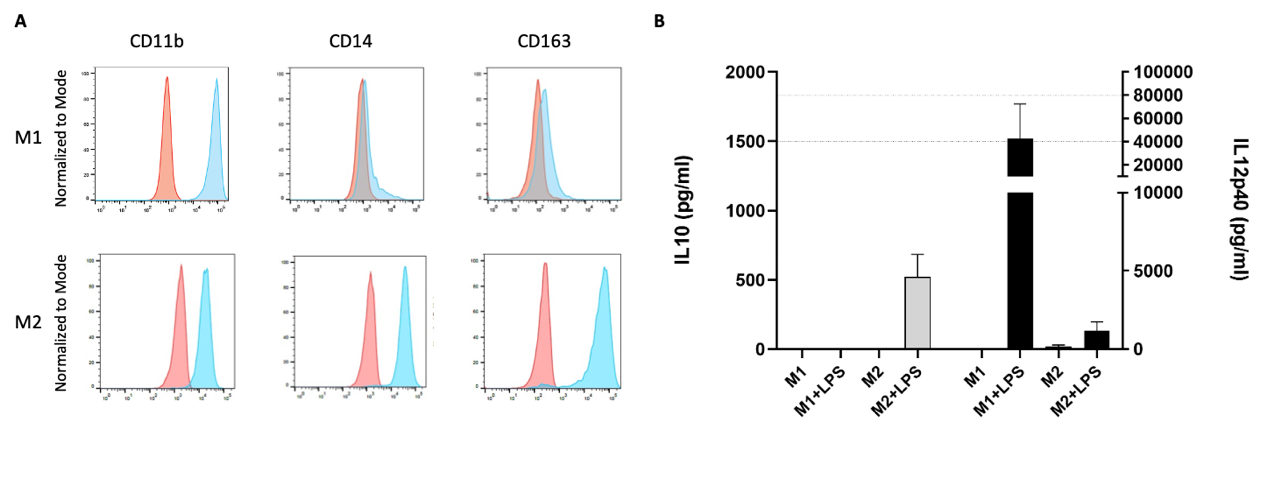


**S1 Fig. Characterization of human M1 and M2 macrophages.** (A) CD11b is relatively high on M1, while CD14 and CD163 are expressed consistently higher on M2 (red: unstained sample; blue: stained sample). (B) After stimulation with LPS, the IL-10 production by M2 is significantly higher than by M1, while M1 secretes high levels of IL-12.


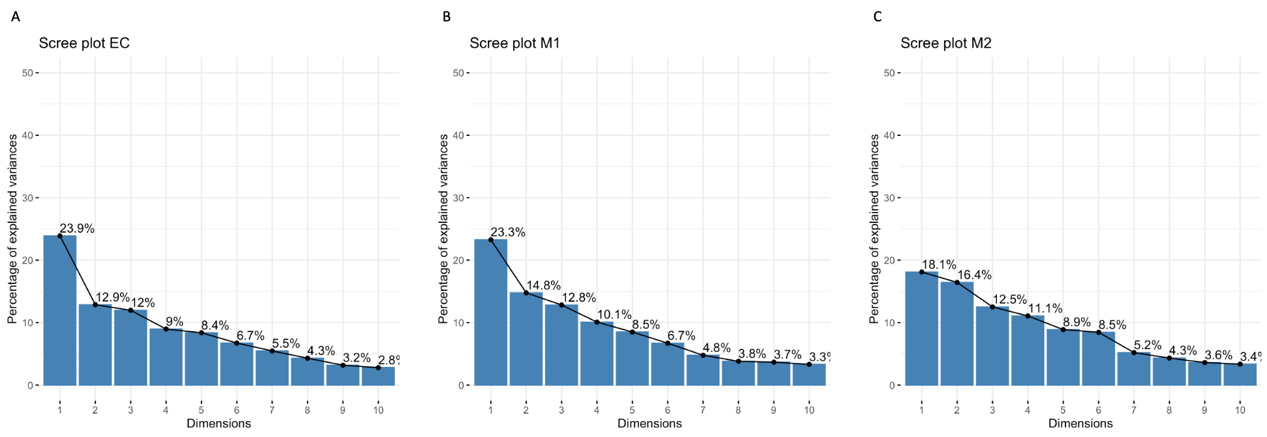


**S2 Fig. Scree plot shows the variance explained by the top 10 principal components in ECs, M1 and M2 after treatment with Ebola VLPs.**


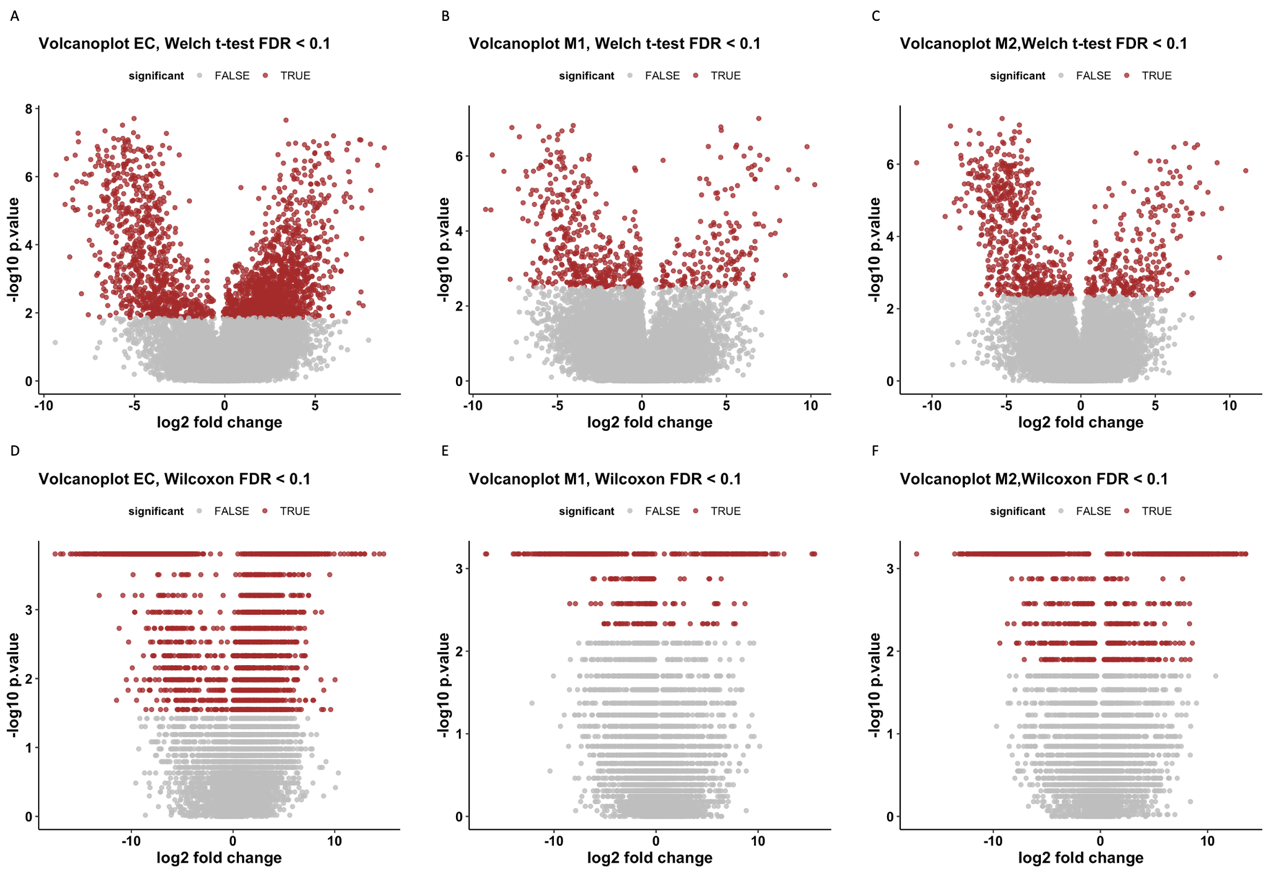


**S3 Fig. Volcano plot of the differentially abundant peaks in ECs, M1 and M2 after treatment with/without Ebola VLPs.** The graphs plot the relative abundance of peaks against their statistical significance, respectively reported as log2 fold change values of the average of mean peak intensity against the respective −log10 adjusted *p*-values, in ECs (A & D), M1 (B & E), and M2 (C & F). Peaks with an adjusted *p*-value of less than 0.1 are shown as significant. Peaks with a log2 fold change less than -1 are attributed to control or treated and peaks with a log2 fold change of greater than 1 are attributed to control or treated.
